# Progressively Discriminative Transfer Network for Cross-Corpus Speech Emotion Recognition

**DOI:** 10.3390/e24081046

**Published:** 2022-07-29

**Authors:** Cheng Lu, Chuangao Tang, Jiacheng Zhang, Yuan Zong

**Affiliations:** 1 Key Laboratory of Child Development and Learning Science (Ministry of Education), Southeast University, Nanjing 210096, China; cheng.lu@seu.edu.cn (C.L.); tcg2016@seu.edu.cn (C.T.); 220194786@seu.edu.cn (J.Z.); 2 School of Information Science and Engineering, Southeast University, Nanjing 210096, China; 3 School of Biological Science and Medical Engineering, Southeast University, Nanjing 210096, China; 4 School of Cyber Science and Engineering, Southeast University, Nanjing 210096, China

**Keywords:** cross-corpus speech emotion recognition, domain adaptation, distribution alignment, discriminative feature learning

## Abstract

Cross-corpus speech emotion recognition (SER) is a challenging task, and its difficulty lies in the mismatch between the feature distributions of the training (source domain) and testing (target domain) data, leading to the performance degradation when the model deals with new domain data. Previous works explore utilizing domain adaptation (DA) to eliminate the domain shift between the source and target domains and have achieved the promising performance in SER. However, these methods mainly treat cross-corpus tasks simply as the DA problem, directly aligning the distributions across domains in a common feature space. In this case, excessively narrowing the domain distance will impair the emotion discrimination of speech features since it is difficult to maintain the completeness of the emotion space only by an emotion classifier. To overcome this issue, we propose a progressively discriminative transfer network (PDTN) for cross-corpus SER in this paper, which can enhance the emotion discrimination ability of speech features while eliminating the mismatch between the source and target corpora. In detail, we design two special losses in the feature layers of PDTN, i.e., emotion discriminant loss $L_{d}$ and distribution alignment loss $L_{a}$. By incorporating prior knowledge of speech emotion into feature learning (i.e., high and low valence speech emotion features have their respective cluster centers), we integrate a valence-aware center loss $L_{v}$ and an emotion-aware center loss $L_{c}$ as the $L_{d}$ to guarantee the discriminative learning of speech emotions except an emotion classifier. Furthermore, a multi-layer distribution alignment loss $L_{a}$ is adopted to more precisely eliminate the discrepancy of feature distributions between the source and target domains. Finally, through the optimization of PDTN by combining three losses, i.e., cross-entropy loss $L_{e}$, $L_{d}$, and $L_{a}$, we can gradually eliminate the domain mismatch between the source and target corpora while maintaining the emotion discrimination of speech features. Extensive experimental results of six cross-corpus tasks on three datasets, i.e., Emo-DB, eNTERFACE, and CASIA, reveal that our proposed PDTN outperforms the state-of-the-art methods.

## 1. Introduction

Emotions reflect the psychological state of human beings, which are usually manifested in physiological and psychological signals [1,2,3,4,5], e.g., facial expression, speech, and electroencephalogram (EEG). As a commonly used communication mean, speech contains rich emotional information. Therefore, making the machine recognize the emotional states of speech, known as the speech emotion recognition (SER) task, is crucial for human–computer interaction (HCI). Generally, the task setting of SER suggests that the training and testing data come from the same corpus, which will cause the trained model on the training data to perform poorly on a new corpus. In recent years, the SER task, which involves training one dataset or several datasets and testing others, i.e., cross-corpus SER, has attracted wide attention.

Since the training and testing data are collected from different datasets, cross-corpus SER faces the issue that the speech samples are quite different in terms of background noise, recording device, language, or speaker, which will lead to the “corpus bias” [6]. Therefore, the cross-corpus SER is more practical than traditional SER tasks. To deal with the bias issue, early works investigated the utilization of low-level descriptors (LLDs) to enhance the emotion discrimination of speech features [7,8]. For instance, Shami et al. [7] firstly implemented the cross-corpus task by using utterance-level acoustical parameters for the naive classifiers, e.g., K-nearest neighbors (KNNs) and support vector machines (SVMs). Further, Schuller et al. [8] defined the cross-corpus SER settings standardly and explored several normalization methods (e.g., speaker normalization, corpus normalization, and speaker-corpus normalization) to reduce the “corpus bias”.

With the rapid development of transfer learning (TL) [9], TL-based methods have shown to be promising for cross-corpus SER [6,10,11,12,13]. TL aims to eliminate the bias between the training and testing data, especially domain adaption (DA) [13,14,15,16], which focuses on the issue of only labeled training data (source domain) and unlabeled testing data (target domain). Motivated by TL, Hassan et al. [17] regarded the dataset difference as a covariance shift, and explored the application of three importance weights (IWs) methods, i.e., kernel mean matching (KMM), unconstrained least-squares importance fitting (uLSIF), and the Kullback–Leibler importance estimation procedure (KLIEP), into the support vector machine (SVM) classifier to reduce this shift and achieved the UAR of 42.7% on FAU Aibo-Mont. Then, Zong et al. [10] proposed a domain-adaptive least-squares regression (DaLSR) method for the corpus shift in cross-corpus SER by projecting speech features to the emotion label space, in which the feature distributions of the training and testing data are as close as possible. The DaLSR obtained the best WAR (52.47%) and UAR (44.41%) for the task of eNTERFACE to Emo-DB at that time. Further, Zhang et al. [13] jointly performed the transfer subspace learning and regression in [10] to learn the corpus-invariant speech features, which achieved the UAR of 49.58% on the task of CASIA to Emo-DB. These works mainly measure the distribution distance of two domains based on the maximum mean discrepancy (MMD). Instead of MMD, Song et al. [6,11,12] also adopted the nearest neighbor graph as the distribution distance metric to reduce domain shifts in the latent space of speech features.

In addition to the subspace learning methods, deep learning methods have also achieved dominating performance as a recent research hotspot. In [18], a domain classifier with a deep neural network (DNN) has been intergraded into the feature extractor and the emotion classifier to learn the emotion-discriminative and domain-invariant feature for cross-corpus SER. Similarly, Abdelwahab et al. [14] also adopted adversarial training to eliminate the domain discrepancy during the common feature learning by an additional domain classifier. Furthermore, Gideon et al. [15] introduced a “meet in the middle” method, i.e., adversarial discriminative domain generalization (ADDoG), to learn the feature of each dataset closer to one another, which can improve the generalization of the dataset representations and then extend it to multiclass ADDoG for the training data with more datasets. The ADDoG obtained the UAR of 0.4749% on the task of MSP-Improv to IEMOCAP.

Although previous works have achieved promising progress in cross-corpus SER, these methods simply consider the SER task as a DA problem, which first learns the common space of speech emotion features for the source and target datasets, and then decreases the distribution distance between the two domains in the common space. In this case, therefore, excessively narrowing the domain distance will impair the emotion discriminativeness of speech features because it is difficult to maintain the completeness of the emotion space only by the emotion classifier (e.g., KNN, SVM, or DNN) [13]. Aiming at this issue, the subspace learning methods perform the sparse constraints (e.g., $\ell_{1}$ norm, $\ell_{2}$ norm, or $\ell_{2,1}$ norm) on the projection matrix of speech features to avoid redundant information for the discriminative emotion feature space [6,10,13]. However, the linear mapping of the subspace learning limits the representation ability of features, which is one of its disadvantages. In addition, the deep learning methods on cross-corpus SER still only consider eliminating the distribution shifts across the source and target domains, while ignoring the preservation of emotion discrimination on speech features.

To address the above issues, we jointly consider the emotion discrimination preservation of speech features and the distribution elimination between the source and target domains, and integrate them into the deep feature extractor. A benefit of this approach is to enhance the emotion discriminativeness of speech while narrowing the distribution discrepancy between two domains such that the emotion-discriminative and domain-invariant speech features can be obtained through the training of a deep end-to-end network.

Therefore, in this paper, we propose a progressively discriminative transfer network (PDTN) for the cross-corpus SER. In the PDTN, we adopt two special losses i.e., emotion discriminant loss $L_{d}$ and distribution alignment loss $L_{a}$, in the high-level feature layers (i.e., fc layers), where $L_{d}$ is combined with the emotion classification loss $L_{ce}$ to enhance the emotion discrimination of speech features and $L_{a}$ decreases the distribution distance of features between the source and target domains. Specifically, $L_{d}$ contains a valence-aware center loss $L_{v}$ and an emotion-aware center loss $L_{c}$, which are inspired by the prior knowledge of speech emotions, i.e., speech emotion features of the high and low valences have their respective cluster centers. Further, we utilize the multi-layer MMD in $L_{a}$ to measure the domain shift of marginal distributions between two domains. The proposed PDTN integrates the three losses, i.e., $L_{a}$, $L_{d}$, and $L_{ce}$ to progressively eliminate the inter-domain discrepancy and improve the emotion discriminativeness of speech features through an end-to-end network training stage. Experimental results on three datasets, i.e., Emo-DB, eNTERFACE, and CASIA, demonstrate the superiority of our proposed PDTN over the comparison methods.

Overall, the contributions of this paper can be summarized as the following three points:

- This paper proposes a novel progressively discriminative transfer network for cross-corpus SER, which jointly considers the two aspects of eliminating the distribution discrepancy across the source and target domains, and enhancing the emotion discrimination of speech features during deep feature learning. Thus, it can avoid the dilemma that previous methods only consider one of two above aspects.
- As far as we know, it is the first work to introduce the prior knowledge of speech emotions, i.e., speech emotion features of high and low valences with their respective cluster centers, into the deep feature learning to enhance the emotion discrimination of speech representations.
- We adopt high-level features of fc layers to perform a practical distribution discrepancy measures under multi-layer features between the source and target domains through a multi-layer MMD metric.

The rest of this paper is organized as follows: Section 2 illustrates the proposed method in detail. Then, we conduct our experiments and discuss the results in Section 3. Finally, Section 4 concludes the paper and gives some points for future research.

## 2. The Proposed Method

**Figure 1:**
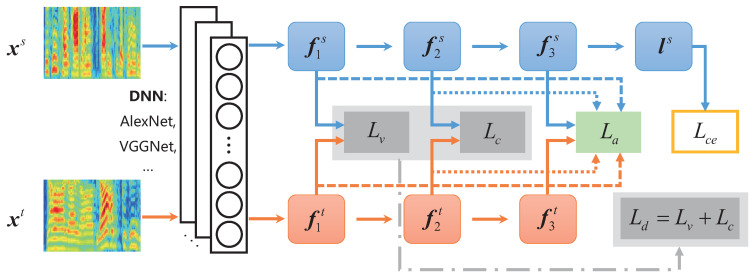
The overview of the progressively discriminative transfer network (PDTN) for cross-corpus SER. PDTN firstly extracts the high-dimensional fc layer features (i.e., $f_{1}$, $f_{2}$, and $f_{3}$) of the source domain data $x^{s}$ and the target domain data $x^{t}$ through DNN (i.e., AlexNet and VGGNet). Then, it uses fc features to calculate the valence-aware center loss $L_{v}$ and emotion-aware center loss $L_{c}$ in emotion discriminant loss $L_{d}$, and distribution alignment loss $L_{a}$, respectively. Finally, it predicts the emotion label of source samples for the emotion classification cross-entropy loss $L_{ce}$.

In this section, we will illustrate the framework of PDTN in detail, shown in Figure 1, which can be divided into three parts, i.e., deep feature extraction, emotion discrimination preservation, and distribution discrepancy elimination.

### 2.1. Deep Feature Extraction

Compared with traditional methods, deep learning has performed well in speech processing, e.g., SER, speech recognition, and speech enhancement. Especially in the SER, DNNs (e.g., CNN and RNN) can extract the high-level feature of speech with more discriminative emotion information. Therefore, we adopt the deep CNN (DCNN) as the backbone network of our proposed PDTN for the deep feature extraction of speech emotion, according to [18,19]. Moreover, as a time–frequency representation of speech, the spectrogram is commonly used for the input feature of DCNN instead of the hand-crafted features.

To illustrate the process of deep feature extraction clearly, firstly, we formalize the labeled training dataset as $D_{s}={\left\{x_{i}^{s},l_{i}\right\}}_{i=1}^{n_{s}}$ and the unlabeled testing dataset $D_{t}={\left\{x_{j}^{t}\right\}}_{j=1}^{n_{t}}$, where $x_{i}^{s}$ and $x_{j}^{t}$ are donated as the spectrogram of the *i*th speech sample in source data and the *j*th sample in source data, $l_{i}$ represents the emotion label of the *i*th speech in source dataset, and $n_{s}$ and $n_{t}$ are the numbers of source and target samples. Notably, since our proposed method is based on unsupervised domain adaptation (UDA) in TL, the target speech samples have no labels.

Then, the spectrogram features of the source and target dataset are fed into the DCNNs to extract the high-level representations of speech emotions. In this paper, we select the AlexNet and VGGNet as the comparison backbones of the proposed PDTN to evaluate the method’s performance. Through the backbones, the spectrograms x are encoded in time and frequency domains by a series of stacked convolutional layers, and further pass through several fully connected (fc) layers to obtain high-dimensional emotional semantic features $f={\left\{f_{k}^{s},f_{k}^{t}\right\}}_{k=1}^{n_{l}}$, where $f_{k}^{s}$ and $f_{k}^{t}$ represent the features of source and target datasets in the *k*th fc layer, and $n_{l}$ is the number of the fc layers. Eventually, the extraction process of the high-level emotion feature $f_{k}=\left[f_{k}^{s},f_{k}^{t}\right]$ in the *k*th fc layer of backbone network $G_{f}\left(\cdot \right)$ can be formalized as

(1) $$\begin{array}{ccc} f_{k} & = & G_{f}\left(\left[x^{s},x^{t}\right];\theta_{f}\right), \end{array}$$

where $\theta_{f}$ is the parameters of the feature extraction network $G_{f}\left(\cdot \right)$. The numbers of fc layers in AlexNet and VGGNet are both set as 3 in this paper.

### 2.2. Emotion Discrimination Preservation

In cross-corpus SER, after extracting the deep speech emotion feature $f_{k}$, the common practice is to either input these high-dimensional features into a fully connected network-based classifier for emotion recognition or to align the distribution of these features both in the source and target domains [14,15,18]. However, since speech emotion is easily disturbed by other factors, e.g., background noise, speaker identity, and language, the emotion features are always confused with the features of these factors [10,11,12]. Therefore, in cross-corpus SER, when the feature distributions between domains are aligned, only utilizing a single emotion classifier cannot effectively disentangle the emotion information from the confusing features in a sufficiently complete feature subspace, which will damage the emotion discrimination of speech features in the feature generalization learning. To address this issue, we introduce an emotion discrimination preservation learning of speech features in the distribution alignment process, which can decouple independent emotion features in the common feature space using the prior knowledge of emotions.

**Figure 2:**
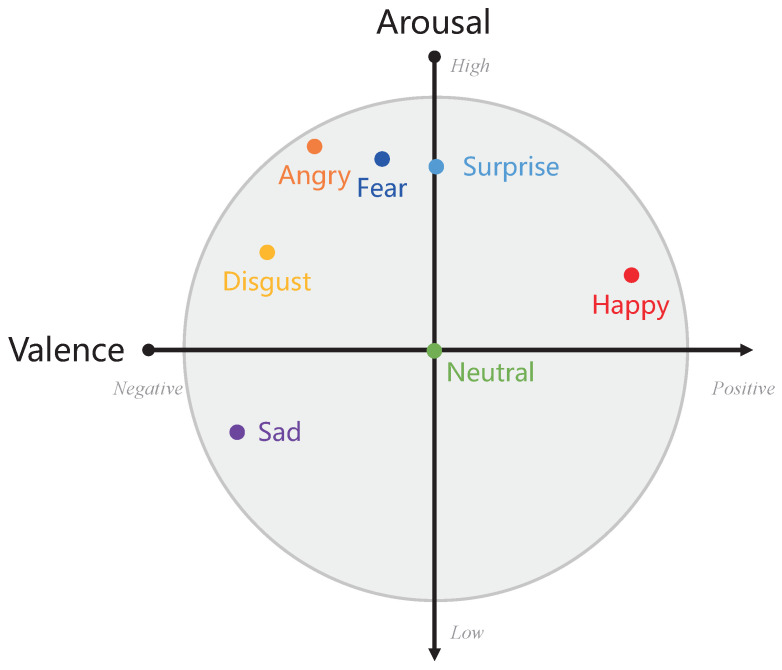
The representation of seven emotions on the 2-dimensional arousal–valence emotion wheel.

As we know, the emotions can be represented on the two-dimensional arousal–valence emotion wheel [20,21,22], shown in Figure 2. It is obvious that each of seven emotions (i.e., *angry, disgust, fear, happy, neutral, sad, and surprise*) have a specific position on the arousal and valence axes of the emotion wheel. According to these positions, the prior knowledge of emotion categories can be indicated, that is, the seven emotions are divided into the negative-valence group (i.e., *angry, disgust, fear, sad, and surprise*), the neutral valence group (i.e., *neutral*), and the positive-valence group (i.e., *happy* and *surprise*) on the valence axis. Under these groups, the emotions in the same group are naturally near each other on the valence, indicating that the centers of their classes are relatively close. On the contrary, the emotions in the different groups have distant centers of emotion classes. Therefore, we introduce the prior knowledge of emotion categories into deep feature learning to maintain the emotion discrimination of speech features.

Specifically, we design a valence-aware center loss $L_{v}$ to model the emotion similarity inside groups and dissimilarity outside groups, which can be donated as

(2) $$\begin{array}{ccc} L_{v} & = & \sum_{i=1}^{n_{b}}max(0,∥f_{k}^{s,i}-v^{l,i}{∥}_{2}^{2}-\alpha_{1})+max(0,\alpha_{2}-∥v_{n}^{b}-v_{p}^{b}{∥}_{2}^{2}), \end{array}$$

where $n_{b}$ is the mini-batch size; $f_{k}^{s,i}$ represents the *k*th fc layer feature of the *i*th speech sample in the source dataset; $v_{n}^{b}$ and $v_{p}^{b}$ are the mini-batch feature centers of the negative-valence emotion group N={angry,disgust,fear,sad,surprise} and the positive-valence emotion group P={neutral,happy}, respectively; $v^{l,i}$ is the feature center of the emotion group where the *l*th class of the *i*th speech sample belongs; and $\alpha_{1}$ and $\alpha_{2}$ are the thresholds to adjust the feature distances within group and between groups, respectively. The feature center of $v^{l,i}$ can be obtained as follows

(3) $$v^{l,i}=\left\{\begin{array}{ccc} v_{n} & , & l_{i}\in N,\\ v_{p} & , & l_{i}\in P, \end{array}\right.$$

where $v_{n}$ and $v_{p}$ are the global centers of the negative-valence and positive-valence emotion groups in the whole source data, which can be calculated during the parameter updating in Algorithm 1. Moreover, the negative-valence feature centers $v_{n}^{b}$ and the positive-valence feature centers $v_{p}^{b}$ in each mini-batch can be denoted as

(4) $$\begin{array}{ccc} v_{n}^{b} & = & \frac{1}{n_{b}^{\prime }}\sum_{\begin{array}{c} 1\leq i\leq n_{b}^{\prime },\\ l_{i}\in N \end{array}}f_{k}^{s,i}, \end{array}$$

(5) $$\begin{array}{ccc} v_{p}^{b} & = & \frac{1}{n_{b}^{\prime \prime }}\sum_{\begin{array}{c} 1\leq j\leq n_{b}^{\prime \prime },\\ l_{j}\in P \end{array}}f_{k}^{s,j}, \end{array}$$

where $n_{b}^{\prime }$ and $n_{b}^{\prime \prime }$ are the numbers of speech samples belonging to N and P in a mini-batch, respectively, and $n_{b}=n_{b}^{\prime }+n_{b}^{\prime \prime }$.

In addition to the arousal-valance-based center loss, we also construct a fine-grained emotion discrimination preservation strategy by fully using the prior information of each emotion category to finely maintain the emotion discriminativeness. Specifically, we design a novel emotion-aware center loss $L_{c}$, which can decrease the inter-class distance and increase the intra-class distance in the source data, represented as follows

(6) $$\begin{array}{ccc} L_{c} & = & \sum_{i=1}^{n_{s}}max(0,∥f_{k}^{s,i}-c^{i}{∥}_{2}^{2}-\alpha_{1})+\sum_{\begin{array}{c} p,q=1,\\ p\neq q \end{array}}^{c}max(0,\alpha_{2}-∥c_{p}^{b}-c_{q}^{b}{∥}_{2}^{2}), \end{array}$$

where $c^{i}$ is the feature center of the emotion category corresponding to the *i*th speech sample in the whole source data, which is implemented for details in Algorithm 1. $\alpha_{1}$ and $\alpha_{2}$ are the thresholds to adjust the distances, respectively. $c_{p}^{b}$ and $c_{q}^{b}$ are the mini-batch feature centers of the *p*th and *q*th emotion category, where $c_{q}^{b}$ can be formalized as

(7) $$\begin{array}{ccc} c_{q}^{b} & = & \frac{1}{n_{b}^{q}}\sum_{1\leq i\leq n_{b}^{q}}f_{k}^{s,i}, \end{array}$$

where $n_{b}^{q}$ is the number of speech samples in a mini-batch corresponding to the *q*th emotion category. The formalization of $c_{p}^{b}$ is similar to $c_{q}^{b}$.

Consequently, we combine $L_{v}$ and $L_{c}$ in deep feature learning to ensure the discrimination of emotions from coarse to fine in the process of distribution discrepancy elimination. Therefore, the loss of emotion discrimination preservation can be represented as

(8) $$\begin{array}{ccc} L_{d} & = & \lambda L_{v}+\gamma L_{c}, \end{array}$$

where λ and γ are the tradeoff parameters to balance the two losses.
**Algorithm 1:** Algorithm for the parameter optimization of PDTN.**Input:** the input features of source and target data: ${\left\{x_{i}^{s}\right\}}_{i=1}^{n_{s}}$, ${\left\{x_{j}^{t}\right\}}_{j=1}^{n_{t}}$;   training labels of source data: ${\left\{l_{i}\right\}}_{i=1}^{n_{s}}$; fc layers: $\left[fc_{1},fc_{2},fc_{3}\right]$;   learning rate: $l_{r}$ and trade-off parameters λ, γ, and μ.**Initialize:**$\theta_{f}$, $\theta_{c}$ randomly.**Output:** the optimized parameters: ${\hat{\theta }}_{f}$, ${\hat{\theta }}_{c}$.**while** the total loss $L_{total}$<ϵ or iter n< maxIter **do**(1) Generate a mini-batch features of source and target data: ${\left\{x_{i}^{s}\right\}}_{i=1}^{n_{b}},\;\;{\left\{x_{j}^{t}\right\}}_{j=1}^{n_{b}};$(2) Extract the high-level features of source and target data: ${\left\{f_{k}^{s},f_{k}^{t}\right\}}_{k=1}^{n_{l}}=G_{f}\left(\left[x^{s},x^{t}\right];\theta_{f}\right);$(3) Calculate the negative-valence and positive-valence feature centers $v_{n}^{b}$ and $v_{p}^{b}$ in each mini-batch by the Equations (4) and (5);(4) Calculate the feature center of *q*th class $c_{q}^{b}$ in each mini-batch by the Equation (7);(5) **if** iter n=1:  Initialize global centers $v_{n}$, $v_{p}$, and $c_{q}$ (or $c_{p}$) in whole source data using steps (4) and (5); **else**:$$\begin{array}{c} ▿v_{n}=\frac{1}{1+n_{b}^{\prime }}\sum_{\begin{array}{c} 1\leq i\leq n_{b}^{\prime },\\ l_{i}\in N \end{array}}\left(v_{n}^{b}-f_{k}^{s,i}\right),v_{n}\leftarrow v_{n}-\eta ▿v_{n},\\ ▿v_{p}=\frac{1}{1+n_{b}^{\prime \prime }}\sum_{\begin{array}{c} 1\leq j\leq n_{b}^{\prime \prime },\\ l_{j}\in P \end{array}}\left(v_{p}^{b}-f_{k}^{s,i}\right),v_{p}\leftarrow v_{p}-\eta ▿v_{p},\\ c_{q}=\frac{1}{1+n_{s}^{q}}\sum_{1\leq i\leq n_{b}^{q}}\left(c_{q}^{b}-f_{k}^{s,i}\right),c_{q}\leftarrow c_{q}-\eta ▿c_{q}; \end{array}$$(6) Calculate $L_{v}$, $L_{c}$, $L_{a}$, $L_{ce}$, and $L_{total}$ using Equations (2), (6), and (10)–(12), respectively;(7) Update the parameter $\theta_{f}$ and $\theta_{c}$:$$\begin{array}{c} \theta_{c}\leftarrow \theta_{c}-\mu \frac{L_{ce}}{\theta_{c}},\theta_{f}\leftarrow \theta_{f}-\mu \frac{L_{total}}{\theta_{f}}; \end{array}$$(8) n=n+1.**end while**

### 2.3. Distribution Discrepancy Elimination

Besides the discriminative feature of emotional speech, another challenge in cross-corpus SER is how to eliminate the domain shift between the source and target data, caused by the factors such as background noise, speaker identity, language, etc. To address this challenge, the moment matching-based methods [12,13] and adversarial learning-based methods [14,15] have been widely investigated and achieved great success. Adversarial learning adopts a domain discriminator to confuse the domain information of features for the discriminative representation of emotional speech, which is prone to a lack of convergence [14]. Moment matching is used to find a suitable metric function to measure the discrepancy between domains, e.g., MMD [13], $\ell_{2}$ distance [23], Deep Coral [24], which is a non-parameter method and easy to implement. Therefore, the previous works of cross-corpus SER mainly integrated MMD into the subspace learning. Nevertheless, the speech emotion features generated by subspace learning are low-level such that it cannot accurately represent the feature distribution of the source and target data, which brings errors to the distance measurement. Thus, in this paper, we utilize the high-level features in fc layers to measure the distribution distance precisely. In addition, since the features of each fc layer correspond to the specific discrimination, inspired by [25,26,27], we also extend the feature alignment of a single layer to a multi-layer adaptation to obtain a more accurate measurement for the domain shift.

Firstly, we implement the distribution discrepancy of the signal layer high-level feature in the *k*th fc layer, namely $D_{k}$, which can be formalized as

(9) $$\begin{array}{ccc} D_{H}^{k} & = & \frac{1}{n_{s}^{2}}\sum_{i=1}^{n_{s}}\sum_{j=1}^{n_{s}}K\left(f_{k}^{s,i},f_{k}^{s,j}\right)\\ & + & \frac{1}{n_{t}^{2}}\sum_{i=1}^{n_{t}}\sum_{j=1}^{n_{t}}K\left(f_{k}^{t,i},f_{k}^{t,j}\right)\\ & - & \frac{2}{n_{s}n_{t}}\sum_{i=1}^{n_{s}}\sum_{j=1}^{n_{t}}K\left(f_{k}^{s,i},f_{k}^{t,j}\right), \end{array}$$

where $k\in [1,2,\ldots ,n_{l}]$, $K\left(f_{k}^{s,i},f_{k}^{s,j}\right)=\langle \phi \left(f_{k}^{s,i}\right),\phi \left(f_{k}^{s,j}\right)\rangle$ is the kernel function in the high-dimension reproducing kernel Hilbert space (RKHS) H, which is denoted as the inner product 〈:,:〉 of the source and target features’ mapping function ϕ.

Further, $D_{H}^{k}$ can be extended to the multi-layer feature distribution distance measurement by integrating the MMD in the two domain features of several fc layers to match the discrepancy between the source and target domains more accurately. Therefore, we can obtain the multi-layer distribution discrepancy distance and take it as the distribution alignment loss $L_{a}$ to constrain the model to gradually eliminate the domain shift between domains during the feature learning process. So, $L_{a}$ can be represented as follows

(10) $$\begin{array}{ccc} L_{a} & = & \frac{1}{n_{s}^{2}}\sum_{i=1}^{n_{s}}\sum_{j=1}^{n_{s}}\prod_{k=1}^{n_{l}}K^{k}\left(f_{k}^{s,i},f_{k}^{s,j}\right)\\ & + & \frac{1}{n_{t}^{2}}\sum_{i=1}^{n_{t}}\sum_{j=1}^{n_{t}}\prod_{k=1}^{n_{l}}K^{k}\left(f_{k}^{t,i},f_{k}^{t,j}\right)\\ & - & \frac{2}{n_{s}n_{t}}\sum_{i=1}^{n_{s}}\sum_{j=1}^{n_{t}}\prod_{k=1}^{n_{l}}K^{k}\left(f_{k}^{s,i},f_{k}^{t,j}\right), \end{array}$$

where $K^{k}$ is the kernel function corresponding to the features in the *k*th fc layer.

### 2.4. PDTN for Cross-Corpus SER

In cross-corpus SER, the spectrograms $x^{s}$ and $x^{t}$ of the source and target data are fed into the backbone network (e.g., AlexNet, VGGNet) to extract the high-level emotion semantic features in the *k*th fc layer, i.e., $f_{k}^{s}$ and $f_{k}^{t}$. After this step, the high-level features in the first fc layer of the source and target data are utilized to calculate the valence-aware center loss $L_{v}$, and the features in the second fc layer are used to generate the emotion-aware center loss $L_{c}$. The combination of $L_{v}$ and $L_{c}$ is regarded as the emotion discrimination preservation loss $L_{d}$ to maintain the emotion information of speech features from coarse to fine. Furthermore, the source feature $f_{3}^{s}$ in the final fc layer is adopted to predict emotion labels for cross-entropy loss $L_{ce}$ by emotion classifier $G_{c}\left(\cdot \right)$, which can be represented as

(11) $$\begin{array}{ccc} L_{ce} & = & \sum_{i=1}^{n_{s}}J\left(G_{c}\left(f_{n_{l}}^{s,i};\theta_{c}\right),l_{i}\right), \end{array}$$

where $\theta_{c}$ denotes the parameter of the emotion classifier $G_{c}\left(\cdot \right)$ and J(·) is the cross-entropy function.

Then, the high-level features in three fc layers of the source and target data are adopted to produce the distribution alignment loss $L_{a}$ for eliminating domain shifts between the source and target domains. Consequently, we can obtain the corpus-invariant and discriminative emotion representation through the total loss $L_{total}$, which can be denoted as

(12) $$\begin{array}{ccc} L_{total} & = & L_{ce}+L_{d}+\mu L_{a}\\ & = & L_{ce}+\lambda L_{v}+\gamma L_{c}+\mu L_{a}, \end{array}$$

where $L_{ce}$ is the cross-entropy loss of the emotion classifier. λ, γ, and μ are all the tradeoff parameters used to balance the different losses.

According to the aforementioned pipeline, the proposed PDTN is optimized by the $L_{total}$ to update the parameters of the backbone network and classifier. The detailed optimization processing is illustrated in Algorithm 1. Thus, in this paper, we utilize three fc layers in both AlexNet and VGGNet backbones, i.e., $fc_{1}$, $fc_{2}$, and $fc_{3}$. Specifically, the features in $fc_{1}$ and $fc_{2}$ are utilized to calculate the $L_{v}$ and $L_{c}$, respectively. The $L_{a}$ is obtained by integrating the features in three fc layers into the alignment loss.

## 3. Experiments

In this section, several experiments are implemented to evaluate our proposed method, and the results are also discussed to illustrate its applicability for cross-corpus SER.

### 3.1. Dataset

- **eNTERFACE** [28] is a public English multi-modal emotion dataset, which contains 1290 audio-visual samples with a sample rate of 48 kHz. In this dataset, six emotions, i.e., *anger, disgust, fear, happiness, sadness*, and *surprise*, are induced by the pre-prepared performance contents. Forty-three volunteers coming from different countries with males and females participated in the recording of the dataset.
- **CASIA** [29] includes 7200 emotional speech sentences with the Chinese language. Each sample is recorded with six emotions, i.e., *anger, fear, happiness, neutral, sadness*, and *surprise*, through some acting contents from four actors containing two males and two females. We utilize 1200 public speech samples with the sample rate of 16 kHz for the experiments.
- **Emo-DB** [30] is collected as a German emotional speech dataset with 535 speech samples by ten native speakers, including five males and five females. In Emo-DB, each sentence is recorded with 16 kHz under seven emotions, i.e., *anger, boredom, disgust, fear, happiness, neutral,* and *sadness*.

In this paper, to perform the cross-corpus SER conveniently, we pick common emotion categories inside two datasets which are adopted for the cross-corpus task. We also design six tasks according to three datasets and the detailed setting is shown in Table 1, in which **e**, **c**, and **b** represent the datasets of eNTERFACE, CASIA, and Emo-DB, respectively.

### 3.2. Experimental Setting

In order to obtain the input of the proposed PDTN, we transform the speech signals to spectrogram features through the short-time discrete Fourier transform (STFT) with the Hamming window, in which the frame length is set as 350, and the FFT points is 1024. It is noted that all speech samples are chosen as the signal channel data and resampled to the sample rate of 16 kHz.

In PDTN, we select the AlexNet [31] and VGGNet (i.e., VGGNet-11) [32] as the backbone networks to evaluate the PDTN’s performance on different networks. In the backbone networks, their three fc layers with the dimensions of 4096, 4096, and class number, i.e., $fc_{1}$, $fc_{2}$, and $fc_{3}$, are adopted to calculate the emotion discrimination preservation loss $L_{d}$ and the distribution alignment loss $L_{a}$. Moreover, to match the input size of backbone networks, the dimension of spectrogram features is resized as 224×224. The implementation of our proposed PDTN is based on the deep learning framework Pytorch with NVIDIA GeForce RTX3090 GPUs and it is optimized by the Adam optimizer [33] with a batch size of 32. Its initialized learning rate is set as 0.0002 with a decay weight of 0.9 and the training epoch is set as 500.

We also describe other parameters for the detail as follows. For instance, we utilize the Gaussian kernel in the MMD of $L_{a}$ and its bandwidth is set according to [34]. For the trade-off parameters, we set the γ and λ by the grid search strategy in the parameter set [0.001, 0.003, 0.005, 0.01, 0.03, 0.05, 0.1, 0.5]. μ is set by an adjusting strategy, which can be formalized as $\mu =\frac{2}{1+e^{-\delta p}}-1$. Then, δ is fixed to 10 and *p* is defined as the ratio of the current number of iterations to the total number of iterations.

In addition, in this paper, we adopt the setting of the cross-corpus SER task by training the PDTN in one dataset (e.g., eNTERFACE) and testing the model in another dataset (e.g., CASIA). Therefore, the six cross-corpus tasks are generated by three datasets, which are summarized as the task section in Table 1. Furthermore, two widely used measure criteria for the recognition accuracy are adopted to evaluate the performance of our proposed PDTN, i.e., the weighted average recall (WAR) and the unweighted average recall (UAR). WAR is denoted as the ratio of the number of correctly predicted samples to the total number of samples, and UAR is the average of the correct rate of each class. UAR has an advantage on measuring the model’s performance on class imbalance databases over WAR. Therefore, combining WAR and UAR can more comprehensively evaluate the performance of PDTN with state-of-the-art methods.

### 3.3. Comparison Methods

To effectively estimate the performance of our proposed PDTN on the cross-corpus SER tasks, we choose several state-of-the-art methods for the comparison, which are described as follows:

- **Baseline methods:** both backbone networks used to extract the high-level features for the experiments.

AlexNet [31]: includes five convolution blocks with the kernel of 5×5 or 3×3 and three fc layers with the dimensions of 4096, 4096, and class number.

VGGNet-11 [32]: consists of eight convolution blocks with the kernel of 3×3 and three fc layers with the dimensions of 4096, 4096, and class number.

- **DA-based methods:** all domain adaptation-based methods for cross-corpus SER tasks by our own implementation.

DAN [27]: contains a deep feature extractor and a domain alignment layer with the MMD in multiple fc layers.

DANN [35]: utilizes the domain adversarial training strategy by a domain discriminator to obtain the task-specific and domain-invariant representation.

Deep CORAL [24]: integrates the CORAL loss based on the second-order statistics (i.e., covariances) into a deep neural network for the end-to-end unsupervised domain adaptation framework.

DSAN [34]: proposes a non-adversarial sub-domain adaptation to align the local distribution discrepancy using joint local MMD.

Note that our proposed PDTN is non-parameterized because the calculation of $L_{c}e$, $L_{d}$, and $L_{a}$ does not require the parameter updating. Therefore, the parameter number of PDTN depends on the backbone networks, i.e., PDTN (AlexNet) has a similar parameter number with AlexNet (60 millions) [31] and the parameter number of PDTN (VGGNet-11) is the same as VGGNet-11 (133 millions) [32]. Furthermore, the parameters of other comparison methods, e.g., DAN, Deep CORAL, and DSAN, also rest with backbone networks. However, DANN has larger parameters than others because of the additional domain discriminator [35]. In addition, compared with AlexNet, VGGNet-11, and DANN, the proposed PDTN, DAN, Deep CORAL, and DSAN all design novel lossless resulting in extra computational complexity. Specifically, PDTN, DAN, and DSAN are based on MMD ($O(n^{2})$) and Deep CORAL is based on the second-order covariance ($O(n^{4})$), where *n* is the larger one of source number $n_{s}$ and target number $n_{t}$.

### 3.4. Results and Discussions

The experimental results of six cross-corpus SER tasks are reported in Table 2 with WAR and UAR. The comparison results reveal that our proposed PDTN based on the two backbone networks, i.e., AlexNet and VGGNet-11, can achieve the best performance over other state-of-the-art methods. In detail, the DA-based methods are superior to the baseline methods for all six tasks of cross-corpus SER on the average accuracies. For each task, the DA-based methods also surpassed the performance of most tasks. Significantly, the discrepancy-based methods, i.e., DAN and Deep CORAL, achieve the comparable recognition rate with the adversarial-based method, i.e., DANN, demonstrating that the distribution alignment strategy, either distance measurement or adversarial training, can promote the corpus-invariant emotion features. Furthermore, DSAN has better performance than these three DA-based methods due to the sub-domina alignment strategy taken into account in DSAN. Furthermore, our proposed method goes beyond the mentioned DA-based methods. This is because the proposed PDTN framework not only adapts the marginal distribution between multiple layers but also maintains the emotion discriminative of speech features.

From the results in the Table 2, we can also observe that the tasks of b→e, e→c, and c→e have worse performances than other tasks (i.e., e→b, b→c, and c→b). This situation indicates that variations in training and test datasets may affect the generalization performance of all cross-corpus methods. In addition, it is also interesting to find that the actuaries of b→e are less than e→b, which may be because the database of Emo-DB is small such that it cannot sufficiently obtain robust speech emotion features. Furthermore, neither c→e nor e→c perform promisingly. This is very likely because the CASIA and eNTERFACE are based on different languages, as CASIA is a Chinese dataset and eNTERFACE is an English one. The disparities across languages lead to the emotion variations in speech, which is also a research hotspot in the field of SER. Nevertheless, our proposed PDTN outperforms both the average accuracies and the performance of each task, demonstrating the superiority of the PDTN.

### 3.5. Ablation Experiments

To verify the effects of different components in the proposed PDTN, we also conduct the ablation study to illustrate this point through extensive experiments. The results with WAR and UAR for ablation experiments are illustrated in Table 3, in which PDTN_S and PDTN_M represent the signal-layer and multi-layer distribution alignment strategy in the PDTN framework according to Section 2.3. Furthermore, we select several key components of PDTN to explore their functions for cross-corpus SER. For instance, PDTN_M w/o $L_{c}\;\&\;L_{v}$, PDTN_M w/o $L_{v}$ denote the model under the PDTN framework without the $L_{c}$ and $L_{v}$ losses and the one without the $L_{v}$ loss, respectively. Thus, for convenient comparison purposes, we adopt VGGNet-11 as the backbone network of PDTN for the ablation study. Thus, the PDTM_M herein is the proposed PDTN (VGGNet-11) in Table 2.

From the ablation results in Table 3, firstly, it is clear that PDTN_M w/o $L_{c}\;\&\;L_{v}$ outperforms PDTN_S w/o $L_{c}\;\&\;L_{v}$ in terms of the average accuracies, which indicates that the multi-layer alignment can obtain more domain-invariant features of speech emotions. Secondly, the performances of PDTN_M w/o $L_{c}\;\&\;L_{v}$ and PDTN_M w/o $L_{v}$ demonstrate that the emotion-aware loss $L_{c}$ we designed in the PDTN framework could facilitate the speech emotion feature learning with more discrimination. Thirdly, the PDTN achieves the best performance compared to other ablation components in the average accuracies. Moreover, PDTN illustrates its superior recognition rates in most of cross-corpus SER tasks except c→e. These comparison results all demonstrate our proposed emotion discrimination preservation loss $L_{d}$, including the valence-aware loss $L_{v}$ and emotion-aware loss $L_{c}$, and distribution alignment loss $L_{a}$ can obtain more discriminative and corpus-invariant representations of emotional speech.

### 3.6. Visualization for Feature Distribution

**Figure 3:**
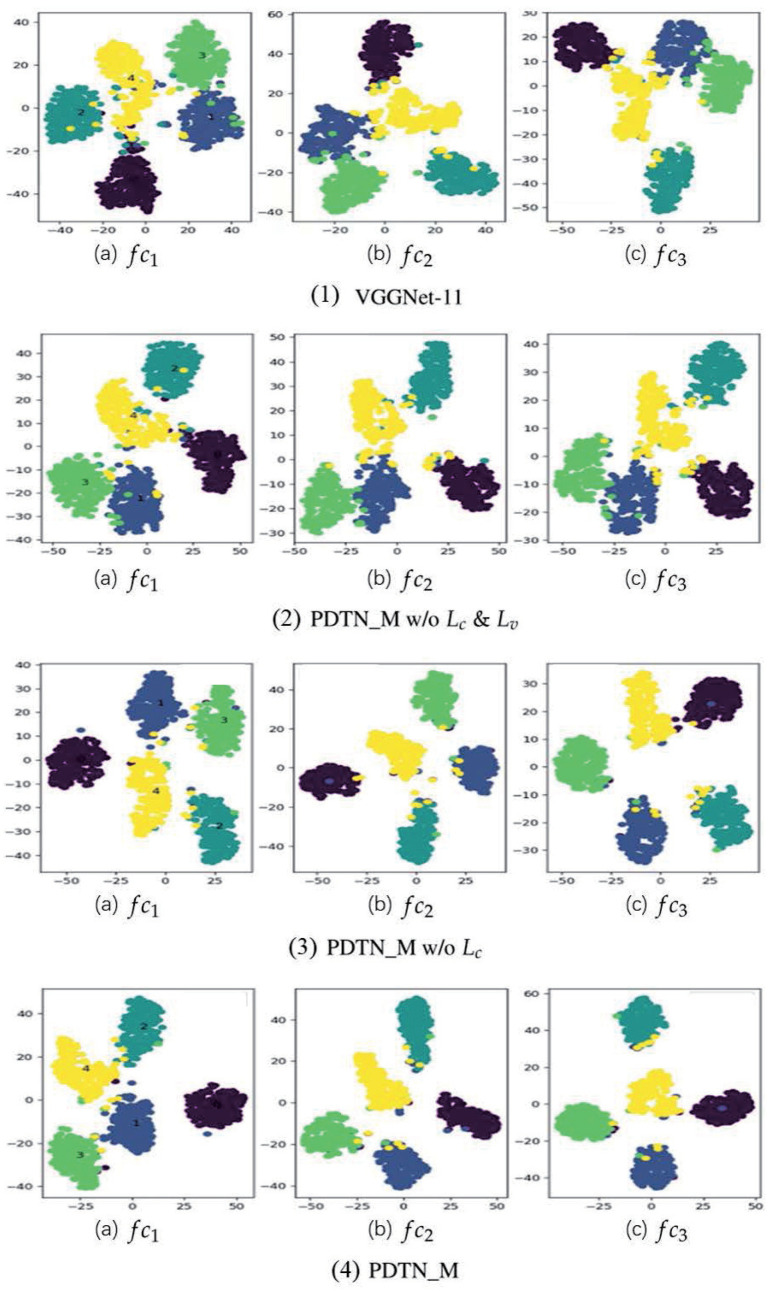
The visualization of feature distributions under different emotions generated by three fc layers (i.e., $fc_{1}$, $fc_{2}$, and $fc_{3}$) of PDTN (VGGNet-11) for the task of e→b.

The key to copying with cross-corpus SER is to extract the discriminative speech emotion feature. Therefore, to demonstrate the superiority of the proposed method on emotion discriminative preservation, we choose the features under the task of e→b for the visualization. The feature distributions of different emotions are visualized in Figure 3, in which the features are generated by the fc layers (i.e., $fc_{1}$, $fc_{2}$, and $fc_{3}$) in the PDTN based on VGGNet-11. The distributions are shown through t-SNE [36], and the points of different colors represent the corresponding emotions, i.e., *anger*, *disgust*, *fear*, *happiness*, and *sadness*.

The sub-figures from (a)–(c) of Figure 3(1)–(3) illustrate that the deeper the fc layer, the more compact the distribution margin of each emotion, indicating that the deeper fc layer features contain stronger emotion discrimination. In addition, from Figure 3(1)–(3), we can also observe that, with the integration of distribution alignment loss $L_{a}$, emotion-aware loss $L_{c}$, and valance-aware loss $L_{v}$, the features in three fc layers become more dispersed among different emotions, and more compact among the same emotion. These visualizations all demonstrate that our proposed PDTN framework is adept at maintaining the emotion discrimination of speech features while eliminating the distribution shift between training and testing data.

## 4. Conclusions

In the paper, we propose a progressively discriminative transfer network (PDTN) for cross-corpus SER, aiming at preserving the emotion discrimination of speech emotion features and eliminating distribution discrepancy between the training and testing data. In PDTN, we design the special discriminative loss $L_{d}$ based on the prior knowledge of speech emotions, including the valence-aware loss $L_{v}$ and emotion-aware loss $L_{c}$, to assist the emotion classifier in enhancing the discrimination of speech features in deep feature learning processing. Then, we also adopt the multi-layer distribution alignment based on MMD to reduce the domain shifts between the source and target data. The experimental results of six cross-corpus SER tasks on three public datasets (i.e., Emo-DB, eNTERFACE, and CASIA) show that our proposed PDTN can obtain the more discriminative and domain-invariant representation of emotional speech than the state-of-the-art methods. In fact, the distance metric we adopt is based on the marginal distribution. Therefore, we will explore integrating conditional distribution to obtain a finer-grained measure for the domain shift in the future.

## Figures and Tables

**Table 1:** Data statistics of six cross-corpus SER tasks on three public datasets, where **e**, **c**, and **b** represent eNTERFACE, CASIA, and Emo-DB, respectively.

Task	Dataset (# Total Number)	Emotion Category (# Samples of Each Emotion)
**b** →**e**, **e** → **b**	**b** (375)	*anger* (127), *disgust* (46), *fear* (69), *happiness* (71), *sadness* (62)
	**e** (1052)	*anger* (211), *disgust* (211), *fear* (211), *happiness* (208), *sadness* (211)
**b** →**c**, **c** → **b**	**b** (408)	*anger* (127), *fear* (69), *happiness* (71), *neutral* (79), *sadness* (62)
	**c** (1000)	*anger* (200), *fear* (200), *happiness* (200), *neutral* (200), *sadness* (200)
**c** → **e**, **e** → **c**	**c** (1052)	*anger* (200), *fear* 200, *happiness* (200), *sadness* (200), *surprise* (200)
	**e** (1000)	*anger* (211), *fear* (211), *happiness* (208), *sadness* (211), *surprise* (211)

**Table 2:** The experimental results (WAR/UAR[%]) compared with the state-of-the-art methods on CASIA, eNTERFACE, and Emo-DB for cross-corpus SER tasks, where the best results are highlighted in bold.

Method	e → b	b → e	b → c	c → b	e → c	c → e	Average
AlexNet [31]	42.40/31.03	29.56/29.49	32.90/32.90	43.13/42.23	27.60/27.60	26.33/26.30	33.65/31.59
VGGNet-11 [32]	44.26/43.23	30.70/30.70	35.10/35.10	44.36/38.95	28.80/28.80	29.65/29.60	35.48/34.40
DAN [27]	49.82/40.41	36.12/36.13	39.00/39.00	50.98/49.85	29.00/29.00	31.46/31.47	39.89/37.64
DANN [35]	52.80/43.68	33.27/33.38	39.20/39.20	54.16/53.71	29.80/29.80	29.24/29.25	39.62/38.05
Deep CORAL [24]	53.07/43.38	35.07/35.03	38.30/38.30	50.73/48.28	31.00/31.00	30.89/30.89	39.84/37.81
DSAN [34]	52.16/46.90	36.29/36.25	40.30/40.30	51.81/50.69	29.70/29.70	32.61/32.61	40.47/39.41
PDTN (AlexNet)	54.60/47.12	38.30/38.32	42.80/42.80	57.59/57.21	35.10/35.10	35.50/**35.50**	43.99/ 42.70
PDTN (VGGNet-11)	**56.80/54.48**	**38.49/38.60**	**44.70/44.60**	**62.01/61.65**	**35.20/35.20**	**35.74**/35.43	**45.49/44.99**

**Table 3:** Ablation experiments of PDTN based on the VGGNet-11 backbone network, where the best results (WAR/UAR[%]) are highlighted in bold.

Method	e → b	b → e	b → c	c → b	e → c	c → e	Average
PDTN_S w/o $L_{c}\;\&\;L_{v}$	52.80/50.40	35.83/35.81	40.20/40.20	55.39/54.85	34.10/34.10	34.03/33.97	42.05/41.54
PDTN_M w/o $L_{c}\;\&\;L_{v}$	53.00/51.07	36.31/36.36	41.60/41.50	58.33/54.55	33.80/33.80	34.88/34.77	42.99/42.01
PDTN_M w/o $L_{v}$	54.44/51.56	38.02/37.97	44.00/43.90	59.06/58.66	34.60/34.60	**35.93/35.67**	44.34/43.31
PDTN_M	**56.80/54.48**	**38.49/38.60**	**44.70/44.60**	**62.01/61.65**	**35.20/35.20**	35.74/35.43	**45.49/44.99**
PDTN_S	54.66/51.87	36.43/36.32	44.40/44.40	57.84/ 56.53	34.50/34.50	35.45/35.14	43.88/43.12

## Data Availability

Not applicable.
